# Bioevaluation of superparamagnetic iron oxide nanoparticles (SPIONs) functionalized with dihexadecyl phosphate (DHP)

**DOI:** 10.1038/s41598-020-59478-2

**Published:** 2020-02-17

**Authors:** Adam Aron Mieloch, Magdalena Żurawek, Michael Giersig, Natalia Rozwadowska, Jakub Dalibor Rybka

**Affiliations:** 1 Center for Advanced Technology, Adam Mickiewicz University in Poznan, Uniwersytetu Poznańskiego 10, 61-614 Poznan, Poland; 2 Institute of Human Genetics, Polish Academy of Sciences, Strzeszynska 32, 60-470 Poznan, Poland; 3 Department of Physics, Institute of Experimental Physics, Freie Universität, Arnimallee 14, 14195 Berlin, Germany

**Keywords:** Nanoparticles, Nanoparticles

## Abstract

Superparamagnetic iron oxide nanoparticles (SPIONs) have been investigated for wide variety of applications. Their unique properties render them highly applicable as MRI contrast agents, in magnetic hyperthermia or targeted drug delivery. SPIONs surface properties affect a whole array of parameters such as: solubility, toxicity, stability, biodistribution etc. Therefore, progress in the field of SPIONs surface functionalization is crucial for further development of therapeutic or diagnostic agents. In this study, SPIONs were synthesized by thermal decomposition of iron (III) acetylacetonate Fe(acac)_3_ and functionalized with dihexadecyl phosphate (DHP) *via* phase transfer. Bioactivity of the SPION-DHP was assessed on SW1353 and TCam-2 cancer derived cell lines. The following test were conducted: cytotoxicity and proliferation assay, reactive oxygen species (ROS) assay, SPIONs uptake (*via* Iron Staining and ICP-MS), expression analysis of the following genes: alkaline phosphatase (*ALPL*); ferritin light chain (*FTL*); serine/threonine protein phosphatase 2A (*PP2A*); protein tyrosine phosphatase non-receptor type 11 (*PTPN11*); transferrin receptor 1 (*TFRC*) *via* RT-qPCR. SPION-DHP nanoparticles were successfully obtained and did not reveal significant cytotoxicity in the range of tested concentrations. ROS generation was elevated, however not correlated with the concentrations. Gene expression profile was slightly altered only in SW1353 cells.

## Introduction

Utilization of superparamagnetic iron oxide naoparticles (SPIONs) spans from diagnostics, imaging and magnetic separation to targeted drug delivery and magnetic hyperthermia^1–4^. This chemically inert and biocompatible material provides a great platform for biomedical applications. The polydispersity level is a crucial factor affecting many properties of SPIONs such as e.g. magnetic properties, biodistribution, cytotoxicity^5^. The most robust methods of SPIONs synthesis, providing highly monodisperse particles at large quantities, rely on thermal decomposition of organic salts. The drawback of these methods is the need to utilize surfactants during the synthesis, which are indispensable for a proper particle formation. Consequently, the surface of as-obtained SPIONs is covered with hydrophobic moieties and requires  subsequent functionalization. This step does not only provide hydrophilic properties, necessary for many biological applications, but also allows for fine tuning of such properties as e.g. surface charge, hydrodynamic radius, colloidal stability etc. which in turn affect their overall performance. Numerous studies have demonstrated different types of ligands used for iron oxide nanoparticles` functionalization. Some of them include: poly(ethylene glycol)(PEG), poly(vinyl pyrrolidone)(PVP), poly(vinyl alcohol) (PVA), poly(lactic-co-glycolic acid) (PLGA), dextran, gelatin, starch, alginate, chitosan, albumin, casein, polydopamine, dendrimers and many more^6–17^. However, there is scarcity of data regarding functionalization exposing phosphate group. From the standpoint of bioactivity and biodistribution, physicochemical properties of nanoparticles play a crucial role in protein corona formation. Upon exposition to biological fluids, such parameters as e.g. size, surface curvature, surface charge, hydrodynamic diameter or functional groups, govern the affinity of certain proteins to the nanoparticles‘ surface^18^. Moreover, differences in the protein corona composition have been demonstrated to correlate with SPIONs biodistribution *in vivo*^19^. The aim of this study is to provide a detailed protocol for SPIONs functionalization with dihexadecyl phosphate (DHP) and broad biological assessment of its interactions with human cells. The following biological analyses of the SPION-DHP were performed: proliferation/viability assay, iron content measurements, reactive oxygen species (ROS) generation, gene expression profile including: alkaline phosphatase (ALPL); ferritin light chain (FTL); serine/threonine protein phosphatase 2A (PP2A); protein tyrosine phosphatase non-receptor type 11 (PTPN11); transferrin receptor 1 (TFRC).

## Results

### Dihexadecyl (DHP) functionalization

**Figure 1:**
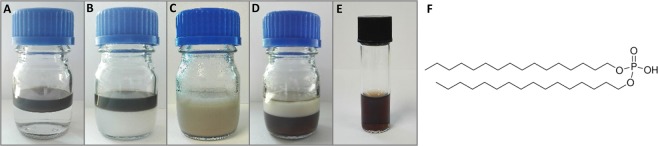
Steps of SPIONs functionalization with DHP. (**A**) Upper phase: hexane, DHP, iron oxide nanoparticles. Lower phase: water. (**B)** Solution after phase transfer of DHP from hexane to water. (**C**) Solution after functionalization. (**D**) 12 h after functionalization. (**E)** Functionalized iron oxide nanoparticles after purification. (**F)** molecular structure of dihexadecyl phosphate (DHP).

**Figure 2:**
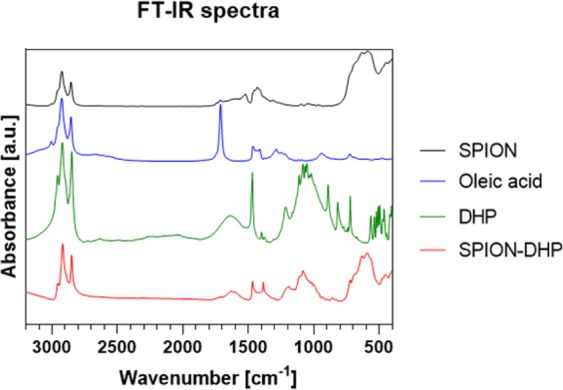
Fourier Transform Infrared Spectroscopy analysis.

The surface of as synthesized nanoparticles is covered in oleic acid residues, with hydrophobic chain directed outward, into the solution. To obtain solubility in water, 15.6 ± 0.9 nm superparamagnetic iron oxide nanoparticles (Fig. S1) were functionalized with DHP surfactant, containing two hydrophobic chains and hydrophilic phosphate group. Functionalization is achieved through hydrophobic interactions of DHP and oleic acid alkyl chains. In the first step, DHP is dissolved in hexane and mixed with chloroform solution of iron oxide nanoparticles (Fig. 1A). In the next step, water is added and the solution is mixed until water phase becomes turbid. This stage of DHP phase transfer from hexane to water was crucial to successful functionalization (Fig. 1B). Post synthesis, solution was uniformly light brown with layer of foam at the top (Fig. 1C). 12 h incubation after synthesis allows for phase separation and removal of the unbound DHP located at the boundary between water and hexane and partially dispersed in hexane (Fig. 1D). After magnetic separation and filtration, sample was redispersed in water and subjected to analyses (Fig. 1E). In order to confirm that the functionalization was successful, FT-IR analysis was performed (Fig. 2). The concentration of SPION-DHP was measured with TGA, described in material and methods. In our previous studies, hydrodynamic radius (*via* dynamic light scattering – DLS) and ζ-potential of DHP coated SPIONs was assessed^20^. In the study: ζ-potential = −44.0 ± 3.4 mV and hydrodynamic diameter = 53.75 ± 1.93 nm.

FT-IR analysis confirmed the presence of DHP on the surface of SPIONs. SPION-DHP clearly shows band patterns corresponding to all constituents (Table 1). Bands at 3000–2800 cm^−1^ relate to CH_2_ and CH_3_ groups of oleic acid and DHP. The signal between 1700–1600 cm^−1^ stems from O=P-H group characteristic for DHP. The wide peak at 1415–1085 cm^−1^ is the most prominent signal derived from P=O groups^21,22^. The signal at around 600 cm^−1^ corresponds to Fe-O bonds^23^.

**Table 1:** Fourier Transform Infrared Spectroscopy spectra characterization.

Absorption [cm^−1^]	Group
3000–2800	CH_2,_ CH_3_
1760	C=O, carboxylic acid
1690	C=C, isolated
1740–1600	O=P-OH
1415–1085	P=O
1260–1000	C-O
1040–909	P-O
600	Fe-O

### Viability/proliferation analysis

**Figure 3:**
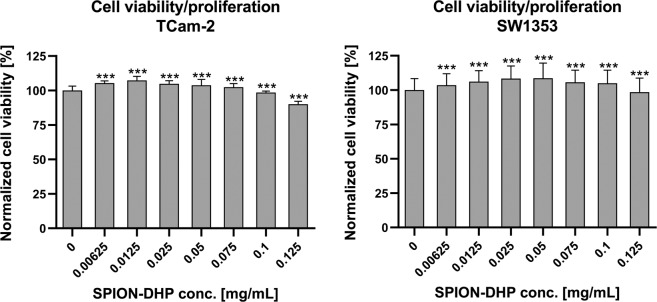
CellTiter-Glo 2.0 assay. One-way ANOVA statistical analysis was used to calculate the significance of the data, α = 0.05. 24 h after seeding, SPION-DHP at different concentrations were added and incubated for 24 h. Asterisks indicate statistically significant differences in comparison to control group (***P ≤ 0.05).

Cell viability/proliferation assay indicates very low cytotoxicity in the range of tested concentration (Fig. 3). At the highest concentration of SPION-DHP (0.125 mg/mL) the viability of TCam-2 was 90 ± 2%; SW1353 98 ± 10%, normalized for the cells without nanoparticles.

### Iron staining

**Figure 4:**
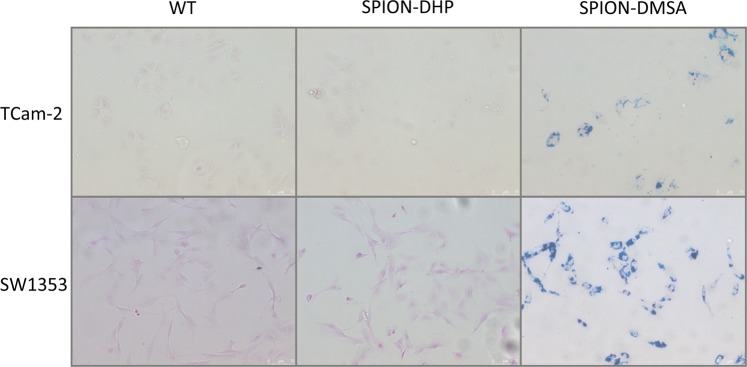
Prussian blue staining was performed for quantitative evaluation of the SPIONs uptake using the Iron Staining Kit (Sigma Aldrich, MO, USA). TCam-2 and SW1353cells were seeded at density of 50,000 and 100,000 cells (respectively) per well on 12-well plate. After 24 h incubation the SPION-DHP and SPION-DMSA were applied to cells in complete growth medium to final concentration of 0.1 mg/mL. Prussian blue staining was performed 24 h after iron oxide nanoparticles administration.

**Figure 5:**
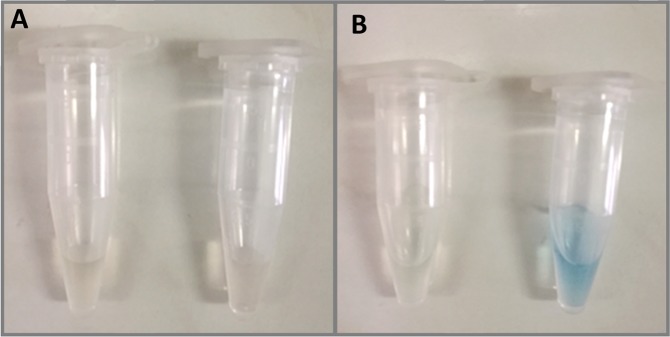
SPION-DHP and SPION-DMSA in Prussian blue reaction. (**A**) SPION-DHP (left) and SPION-DMSA (right) at 0.1 mg/mL concentration in PBS. (**B**) SPION-DHP (left) and SPION-DMSA (right) after 10 minutes incubation in Potassium Ferrocyanide and Hydrochloric Acid Solution (IRON STAIN Solution, Sigma Aldrich, MO, USA). Ionic iron of DMSA-NP reacted with acid ferrocyanide producing a blue color.

**Figure 6:**
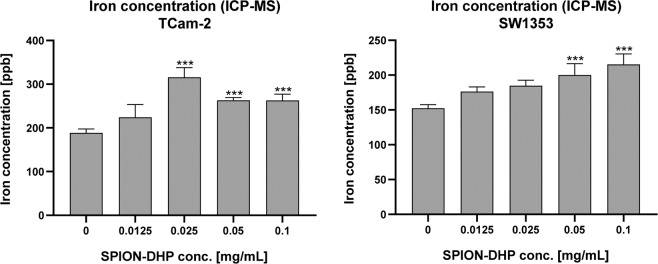
Inductively Coupled Plasma Mass Spectrometry (ICP-MS) analysis. One-way ANOVA statistical analysis was used to calculate the significance of the data, α = 0.05. 24 h after seeding, SPION-DHP at different concentrations were added and incubated for 24 h. Asterisks indicate statistically significant differences in comparison to control group (***P ≤ 0.05).

Prussian blue-based staining was performed to assess the cellular internalization of SPION-DHP. However, no staining was detected for both cell types. For comparison, SPION-DMSA nanoparticles were administered (ζ = −49,3 mV^24^). In this case, blue stain was observed, indicating internalization of the nanoparticles (Fig. 4). In order to establish if DHP coated nanoparticles were not internalized or if they can’t be detected with Prussian blue staining, 0.1 mg/mL of both SPION-DHP and SPION-DMSA were suspended in PBS, and subjected to the reaction (Fig. 5). No reaction was detected for SPION-DHP, indicating Prussian blue staining incompatible for the assessment of cellular iron concentration for DHP funcionalized SPIONs. To confirm the internalization of SPION-DHP, Inductively Coupled Plasma Mass Spectrometry (ICP-MS) analysis was performed instead (Fig. 6). The method was developed and described in our previous study^24^. For TCam-2 cells, the highest intracellular iron concentration (316 ± 18 ppb) was detected at 0.025 mg/mL of SPION-DHP. For SW1353, the highest concentration (215 ± 15 ppb) of intracellular iron was detected for the highest concentration of SPION-DHP, 0.1 mg/mL.

### ICP-MS iron concentration analysis

#### Reactive oxygen species (ROS) generation

**Figure 7:**
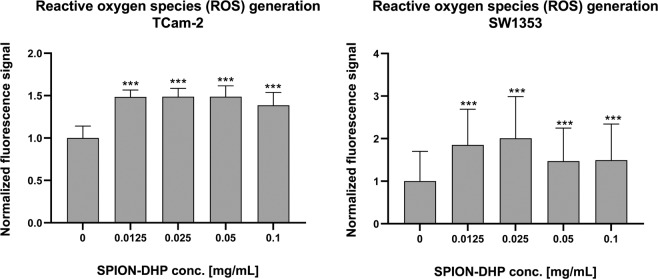
Reactive Oxygen Species (ROS) test. ROS production in SPION-DHP treated cells was investigated using fluorogenic probes DCFDA/H2DCFDA - Cellular ROS Assay Kit One-way ANOVA statistical analysis was used to calculate the significance of the data, α = 0.05. 24 h after seeding, SPION-DHP at different concentrations were added and incubated for 24 h. Asterisks indicate statistically significant differences in comparison to control group (***P ≤ 0.05).

ROS generation was assessed using fluorogenic probes DCFDA/H2DCFDA (Fig. 7). Although slight, statistically significant increase was observed for both TCam-2 and SW1353 after SPION-DHP administration, it was not correlated with the increase of concentration.

#### Gene expression analysis

The following genes where selected for gene expression analysis: transferrin receptor 1 (TFRC); ferritin light chain (FTL); alkaline phosphatase (ALPL); serine/threonine protein phosphatase 2A (PP2A); protein tyrosine phosphatase non-receptor type 11 (PTPN11).

Transferrin receptor (TFRC; CD71). Iron metabolism is crucial for various biochemical processes, providing normal functioning of cells and organs of the human body. Due to its involvement is such processes as e.g. the formation of heme- and iron-containing proteins participating in oxygen transport, energy metabolism, DNA synthesis etc., it requires precise control over its intracellular concentration^25,26^. Transferrin receptor is a membrane glycoprotein, which can import iron by binding a plasma glycoprotein, transferrin (TF). TF is the serum protein with two specific Fe^3+^-binding sites.

At the pH = 7.4, TFRC binds iron-bearing TF, either monoferric or diferric. Subsequently, TF/TFRC assembly is internalized and transferred to endosome, where at the pH = 5.6, iron ions are released^27^. TFRC gene was chosen to investigate if there is a biologically significant iron ions leakage from SPIONs present in cell culture.

Ferritin is an intracellular protein, governing the storage and release of iron ions. Under aerobic conditions, ferritin promotes oxidation of the Fe(III) ions, which are subsequently stored in a form of aggregates. Human ferritin is composed of two subunit types: light chains and heavy chains. The expression of ferritin light chain (FTL) is strictly related to bioavailability of Fe(II) ions^28^. This gene was chosen due to reported increase of ferritin expression after iron oxide nanoparticles administration, which is caused by their gradual degradation, followed by the increased availability of iron ions^29,30^.

Alkaline Phosphatase (ALP; EC: 3.1.3.1) is an abundant glycoprotein bound to the cellular membranes, acting as a potent catalyst of phosphate monoesters hydrolysis at basic pH. The alkaline phosphatases (ALPs) of mammals belong to the category of metalloenzymes. ALPs active site contains two Zn^2+^ and one Mg^2+^ ions, required for enzymatic activity^31^. This gene was chosen on the assumption of interaction between phosphate group from DHP and alkaline phosphate. Despite DHP being phosphate diester, its intracellular hydrolysis could potentially provide the substrate for enzymatic reaction. ALP is encoded by the ALPL gene.

Protein Phosphatase 2A (PP2A; EC: 3.1.3.16) is a crucial and widely expressed serine threonine phosphatase, responsible for the regulation of many cellular processes through the mechanism of dephosphorylation^32^. PP2A is crucial in such processes as e.g.: signal transduction, glycolysis, lipid metabolism, DNA replication, cell proliferation, transcription and translation, cell mobility and apoptosis^33^. Natively, protein phosphatases nucleophilically attack and dephosphorylate three types of amino acids: tyrosine (Tyr), threonine (Thr) and serine (Ser)^34^. This gene was chosen to establish whether DHP can be a potential target for PP2A catalytic activity.

Protein Tyrosine Phosphatase non-receptor Type 11 (PTPN11; EC 3.1.3.48). The product of PTPN11 gene, Src homology region 2 domain-containing phosphatase-2 (SHP-2), is ubiquitously expressed among tissues. SHP2 regulates both physiological and pathological processes including cell survival, migration and proliferation through the positive (signal-enhancing) and/or negative (signal-inhibiting) regulation of signaling pathways. The main SHP2 signaling routes involve phosphatidylinositol 3-kinase (PI3K)-AKT, Ras-Raf-mitogen-activated kinase (MAPK) and Janus tyrosine kinase (JAK)/signal transducer and activator of transcription (STAT) in response to cytokine, hormonal, and growth factor stimulation or genomic damage in a cell-specific manner^35,36^. This gene was chosen to assess if DHP covered SPION can act as a substrate for SHP2 enzymatic activity.

### TCam-2

#### SW1353

**Figure 8:**
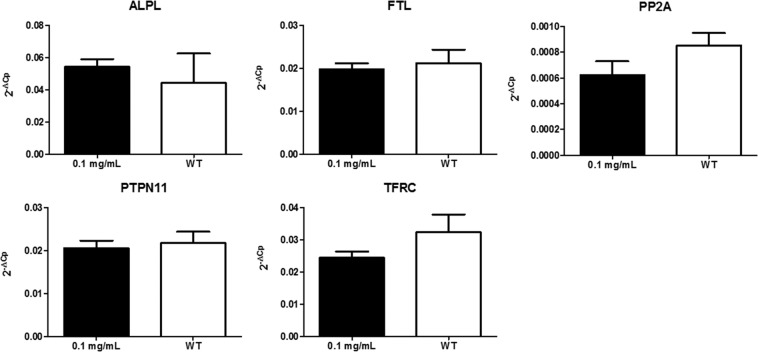
Expression analysis of selected genes in TCam-2 cells treated with SPIONs and wild type. Relative expression level was calculated using the 2−ΔCp formula. The data are presented as mean relative expression level ± SD. TCam-2 cells were treated with 0.1 mg/mL SPIONs; WT- wild type, non-labeled cells; ALPL- alkaline phosphatase; FTL- ferritin light chain; PP2A-serine/threonine protein phosphatase 2A; PTPN11- protein tyrosine phosphatase, non-receptor type 11; TFRC- transferrin receptor 1.

**Figure 9:**
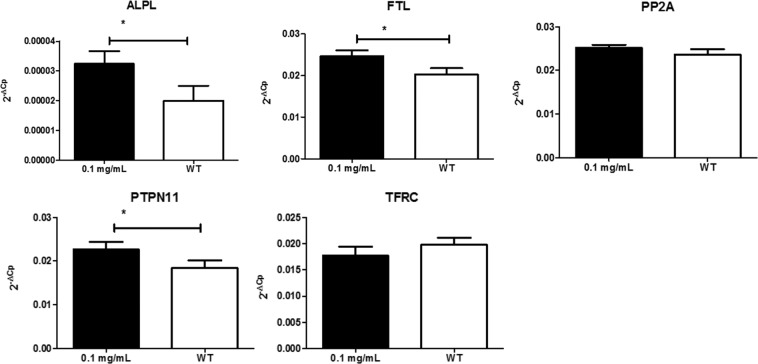
Expression analysis of selected genes in SW1353 cells treated with SPIONs and wild type. Relative expression level was calculated using the 2−ΔCp formula. The data are presented as mean relative expression level ± SD. Asterisk indicate statistical significance (*P < 0.05). SW1353 cells were treated with 0.1 mg/mL SPIONs; WT- wild type, non-labeled cells; ALPL- alkaline phosphatase; FTL- ferritin light chain, PP2A-serine/threonine protein phosphatase 2A; PTPN11- protein tyrosine phosphatase, non-receptor type 11; TFRC- transferrin receptor 1.

No statistically significant changes in all tested genes were observed after administration of 0.1 mg/mL SPION-DHP to TCam-2 cells (Fig. 8). Statistically significant changes in gene expression were observed for ALPL, FTL and PTPN11 in SW1353 cells (Fig. 9).

## Discussion

The functionalization of SPIONs with DHP is a straightforward process, yielding a stable colloidal suspension of SPION-DHP nanoparticles. In our previous studies, these particles were successfully used to create virus-like particles with magnetic cores^20,37^. Viability/proliferation assay revealed very low toxicity up to 0.125 mg/mL concentration for TCam-2 and SW1353 cells (Fig. 3). Iron concentration assay *via* Prussian blue staining revealed that SPION-DHP nanoparticles are resistant to the staining and cannot be detected with this method (Figs. 4 and 5). In comparison, SPION-DMSA nanoparticles were easily recognized with this method. The exact mechanism underlying resistance of SPION-DHP staining against Prussian blue has not been identified. Presumably, DHP coating prevents SPIONs from being dissolved with HCl, which results in lack of iron ions required for Prussian blue reaction. To assess the internalization of SPION-DHP *via* intracellular iron concentration measurement, ICP-MS was used (Fig. 6). In both cases, intracellular iron concentration increased after SPION-DHP administration. For TCam-2, statistically significant increase was observed for all concentrations at maximum of 316 ± 18 ppb for 0.025 mg/mL (188 ± 7 ppb for control). For SW1353, statistically significant increase was observed for 0.05 mg/mL (200 ± 16 ppb) and 0.1 mg/mL (215 ± 15 ppb), in comparison to control (152 ± 5 ppb). This data indicates, that SPION-DHP were successfully internalized by both cell types. Non-linear increase in intracellular iron concentration in TCam-2 cells could be explained if SPION-DHP internalization occurs by receptor-mediated endocytosis. In this case, receptor depletion would inhibit further internalization of the nanoparticles. It has been demonstrated that SPIONs can be internalized by several endocytic uptake pathways, such as phagocytosis, caveolae-dependent endocytosis, clathrin-dependent endocytosis or macropinocytosis^38^. Therefore, cell-type dependent efficiency in SPION-DHP internalization could be assumed. Iron oxide nanoparticles have been shown to facilitate redox reactions (as the reactant or catalyst) resulting in generation of reactive oxygen species. Iron species participate in homogenous Fenton, Fenton-like and Haber-Weiss reactions. Products of these reactions such as superoxide radicals, hydroxyl radicals, or ferryl-oxo complexes are highly reactive in cellular environment, and are capable of exerting severe cellular damage. Additionally, it has been demonstrated that iron oxides can initiate heterogeneous redox reactions at the water/solid interfaces, increasing the probability of ROS-related toxicity^39,40^. Although elevated ROS levels were detected after administration of SPION-DHP to TCam-2 and SW1353 cells, they were not dependent on the concentrations administered (Fig. 7). The observed increase in ROS generation did not have a negative impact on short-term proliferation/viability of the cells. Gene expression analysis revealed discrepancies between cell lines (Figs. 8 and 9). There were no statistically significant changes in gene expression for all genes tested, after administration of 0.1 mg/mL SPOION-DHP in TCam-2 cells. The same concentration elicited increased expression of ALPL, FTL and PTPN11 genes in SW1353 cells. The lack of increase in transferrin receptor suggests that the nanoparticles in culture medium remained intact and did not release significant amounts of iron ions. Slight increase in transferrin light chain expression was observed in SW1353 cells. This may indicate a partial release of iron ions from SPION-DHP nanoparticles. Alkaline phosphatase expression in SW1353 was significantly elevated. However, the lack of the increase in TCam-2 cells indicates that the elevated expression was not a result of DHP hydrolysis, providing substrate for the enzymatic reaction, but rather a complex response for SPION-DHP administration. Protein Tyrosine Phosphatase non-receptor Type 11 expression was also slightly increased in case of SW1353 cells. Similarly to ALP, its involvement in many cellular processes suggests a complex cellular response to the nanoparticles administration rather than a direct interaction with their surface or disintegration products. Biological evaluation of DHP coated SPIONs indicates their low, however cell-type dependent cytotoxicity. TCam-2 cells appeared as more resilient in comparison to SW1353 cells. In regard to protein corona formation, existing studies suggest that upon exposition to biological fluids SPION-DHP nanoparticles would display affinity mainly for albumin and retain its negative surface charge. It has been demonstrated, that regardless of the zeta potential (positive, neutral, negative), polyvinyl acid- or dextran-coated SPIONs, showed negative zeta potential after exposition to PBS + serum solution^41^. However, to elucidate effects of DHP coating on protein corona formation, further studies are required.

### Summary

This study presents a ready-to-use protocol for iron oxide nanoparticles functionalization with dihexadecyl phosphate. SPION-DHP nanoparticles did not reveal significant cytotoxicity in the range of tested concentrations. ROS generation was elevated, however not correlated to the concentrations. Gene expression profile was slightly altered only in SW1353 cells. In summary, this preliminary investigation indicates that SPION-DHP hold a great potential for biological applications.

## Materials and Methods

### Materials

Oleic acid (technical grade 90%), Iron (III) acetylacetonate (97%), Dihexadecyl phosphate, 1-octadecene (90%), 2-butanol (95,5%), Sodium dodecyl sulfate (99%), Paraformaldehyde (95%) were purchased from Sigma-Aldrich (Sigma-Aldrich, MO, USA). Toluene (99,5%), n-Hexane (99%) and Chloroform (98,5%) were purchased from Avantor (Avantor, Gliwice, Poland) were used as received. Water was purified by Hydrolab HLP5 instrument (0.09 µS/cm).

### Synthesis of superparamagnetic iron oxide nanoparticles (SPIONs)

Spherical iron oxide nanoparticles were synthesized via thermal decomposition of iron (III)acetylacetonate Fe(acac)_3_. This method has been described in our previous works^20,42^ Briefly, 6 mmol of Fe(acac)_3_ and 18 mmol of oleic acid were dissolved in 40 mL of 1-octadecene. The reaction was performed with continuous stirring and nitrogen flow. Temperature of the solution was increased to 220 °C and maintained for 1 h. Subsequently, the temperature was increased further to 320 °C and maintained for 1 h. After the synthesis, the solution was left to cool down to ambient temperature and 200 mL of washing solution (3:1 v/v of 2-butanol and toluene) was added. The obtained mixture was placed on a neodymium magnet and left overnight to allow nanoparticles to precipitate. Supernatant was discarded and replaced with fresh washing solution. Sonicating bath was used to resuspend nanoparticles. The washing step was performed thrice. In the final step, nanoparticles were suspended in 20 mL of chloroform. Concentration of the nanoparticles was estimated by dried sample weighing. Size of the particles was analyzed using ImageJ 1.8 Software. 100 particles were analyzed. Mean diameter = 15.6 ± 0.9 nm.

### Transmission electron microscopy (TEM)

10 µl of sample was placed on carbon coated copper grid. The excess was removed with blotting paper. Sample was visualized with Hitachi TEM HT7700 microscope. Images were analyzed with ImageJ software.

### Functionalization with dihexadecyl phosphate (DHP)

DHP functionalization was performed in accordance to our previously described method^20,42^. 10.0 mg of dihexadecyl phosphate were added to 20 mL of hexane and dissolved with heat-assisted magnetic stirring (75 °C, ca. 10 min). After DHP dissolution, a chloroform solution containing 10.0 mg of synthesized iron oxide nanoparticles coated with oleic acid was added. The mixture was shortly sonicated and 80 mL of water were added. Subsequently, the obtained two-phase solution was briefly vortexed and sonicated until the water phase became turbid. In the next step, the solution was placed in a sonicating bath for 3–4 h with no temperature control. After the functionalization, the solution was left overnight to allow for phase separation. The bottom phase was collected and placed near neodymium magnet for 24 h to separate functionalized nanoparticles from the solution. The obtained precipitate was collected, suspended in 2 mL of miliQ water and filtered through 0.22 μm pores. Concentration of the SPION-DHP nanoparticles was measured *via* thermogravimetric analysis described below.

### Functionalization with meso-2,3-dimercaptosuccinic acid (DMSA)

Ligand exchange procedure was performed to exchange the capping ligand from oleic acid to meso-2,3-dimercaptosuccinic acid (DMSA). This method has been described in our previous work^24^. In the first step, 50 mg of DMSA was dissolved in 15 mL dimethylsulfoxide (DMSO) and 100 mg of nanoparticles were diluted in 15 mL of chloroform. Solutions were mixed together and 50 μL of triethylamine was added as the catalyst. Reaction was carried out at 60 °C for 6 hours (shaken vigorously) in a horizontal shaker. Nanoparticles were washed with ethanol, collected with neodymium magnet. The procedure was repeated until the supernatant was transparent, and eventually nanoparticles were resuspended in 20 mL of ethanol. Then, the second step of reaction was carried out. Obtained solution was mixed with 50 mg of DMSA dissolved in 15 mL of DMSO and 50 μL of triethylamine was added. The reaction conditions were the same as in the first step. Washing procedure was also similar, except that ultrapure water was used instead of ethanol. Finally, nanoparticles were resuspended in 10 mL of ultrapure water. For the use in *in vitro* tests, particles were sterile filtered with cellulose acetate syringe filters of two sizes: 0.45 μm and 0.2 μm, respectively.

### Fourier-transform infrared spectroscopy (FT-IR)

The analysis was performed on Bruker FT-IR IFS 66/s spectrometer. The samples were formed into KBr tablets and analyzed in the 4000–400 cm^−1^ range.

### Concentration measurement *via* thermogravimetric analysis (TGA)

Thermogravimetric analysis was performed to measure concentrations of the functionalized SPIONs. This method was performed in accordance to our, previously established, protocol^20^ The analysis was performed on TGA 4000 System (Perkin Elmer apparatus, Waltham, MA, USA). Briefly, a 20 μL sample was taken for measurement. Each sample was measured in triplicate. The sample was heated from 20 to 150 °C at 10 °C/min in nitrogen atmosphere. The lowest mass was taken as fully dried sample and used for further calculations (Fig. S2). The obtained mass was normalized for 20 mg of the initial sample mass. The mean of three measurements was calculated. Density was derived from weighing 5 × 15 μL of the sample and dividing the mean mass by volume.

### Cell culture

SW1353 cells (ATCC® HTB-94™) were cultured in DMEM F12 with Lf-glutamine medium (Lonza, Switzerland), 10% FBS (Sigma Aldrich, MO, USA) and 1% penicillin-streptomycin-amphotericin B (Lonza, Switzerland). TCam-2 cells (kindly gifted from Dr Riko Kitazawa, Department of Diagnostic Pathology, Ehime University Hospital, Matsuyama, Japan) were cultured in RPMI 1640 GlutaMax medium (Gibco, Thermo Fisher Scientific, MA, USA), 10% HyClone FBS (GE Healthcare, IL, USA) and 1% penicillin-streptomycin-amphotericin B (Lonza, Switzerland). All *in vitro* cultures were carried out at 37 °C and 5% CO_2_.

### Inductively coupled plasma mass spectrometry (ICP-MS) analysis

ICP-MS was applied for quantitative determination of superparamagnetic iron oxide nanoparticles (SPIONs) uptake. The analysis was performed on the NexION 300D ICP-MS, Perkin Elmer. SW1353 and TCam-2 cells were seeded at density 6 × 10^4^/cm^2^ and 2 × 10^4^/cm^2^, respectively and cultured for 24 h. Next, the SPIONs were added in culture media to final concentration of 0.1 mg/mL, 0.05 mg/mL, 0.025 mg/mL and 0.0125 mg/mL. The untreated cells were indicated as a control. After 24 h incubation SW1353 and TCam-2 cells (4 × 10^5^ cells) were collected, washed twice with PBS and cell pellets were frozen at −80 °C. Before the analysis, cells were thawed and lysed in 100 μL of 10% SDS. Then, the cell lysates were frozen for 24 h, thawed and placed in sonicating bath for 1 h. 25 µL of cell lysates (1 × 10^5^ cells) were dissolved in 125 µL of 65% nitric acid and shaken at 80 °C for 2 h to remove organic compounds. As-prepared samples were diluted 10 folds in DI water and taken for the measurement. Iron content was determined based on the standard curve prepared with a multi element standard solution for ICP-MS in the 1, 10, 100, 1000 ppb range.

### SPIONs cellular uptake labeling

Prussian blue staining was performed for quantitative evaluation the SPIONs uptake using the Iron Staining Kit (Sigma Aldrich, MO, USA). SW1353 and TCam-2 cells were seeding at density of 1 × 10^5^and 5 × 10^4^ cells (respectively) per well on 12-well plate containing microscope cover glasses. After 24 h incubation the SPION-DHP and SPION-DMSA were applied to cells in complete growth medium to final concentration 0.1 mg/mL, 4 wells per group (respectively). SW1353 and TCam-2 cells with no SPIONs added were used as a control. Prussian blue staining was performed 24 h after iron oxide nanoparticles administration. Cells were washed with PBS and fixed with 4% paraformaldehyde solution in PBS. Subsequently, Prussian blue staining was performed according to the manufacturer’s protocol. In the last step, the cover glasses were mounted onto slide glasses with gelatin solution. SW1353 and TCam-2 cells were visualized with Leica DMi8 microscope.

### Proliferation and cytotoxicity assay

To determine the cytotoxicity of SPIONs and their impact on cells proliferation the CellTiter-Glo 2.0 assay (Promega, WI, USA) was performed according to the manufacturer’s protocol. TCam-2 cells were seeded at density of 2 × 10^4^ cells per well on 96-well microplate (Falcon white/clear bottom plate, Corning, NY, USA), 8 wells per group. After 24 h of culture the SPIONs were applied to cells in growth medium to final concentration 0.125 mg/mL, 0.1 mg/mL, 0.075 mg/mL, 0.05 mg/mL, 0.025 mg/mL, 0.0125 mg/mL and 0.00625 mg/mL. Potential cytotoxicity of SPIONs was tested after 24 h of incubation. CellTiter-Glo Reagent was added in an equal volume (100 µl) to each well. The luminescence was recorded using Tecan Infinite M200 Pro. The SPIONs untreated cells were indicated as a control. Furthermore, the background luminescence was determined in wells containing medium without cells.

### Gene expression analysis

Expression analysis of alkaline phosphatase (*ALPL*); ferritin light chain (*FTL*); serine/threonine protein phosphatase 2 A (*PP2A*); protein tyrosine phosphatase non-receptor type 11 (*PTPN11*); transferrin receptor 1 (*TFRC*) genes was evaluated by quantitative reverse-transcription PCR (RT-qPCR). Total RNA from wild type and 0.1 mg/mL SPIONs treated SW1353 and TCam-2 cells was isolated using RNeasy Mini kit (Qiagen, Hilden, Germany) according to the manufacturer’s protocol. The quality and quantity of RNA was estimated by spectrophotometric measurements (NanoDrop ND1000, Thermo Scientific, MA, USA) and 1% agarose gel electrophoresis. 500 ng of RNA was used in RT reaction performed with QuantiTect Reverse Transcription kit (Qiagen, Hilden, Germany). qPCR was evaluated using 2 µl of diluted 1:10 cDNA samples, 1x Hot FIREPol EvaGreen qPCR Mix (Solis, BioDyne, Tartu, Estonia) and 150 nM forward, reverse primers in a total volume of 20 µl. The primer sequences were obtained from the University of California Santa Cruz Genome Browser on Human Genome hg19 assembly (https://genome.ucsc.edu/), Primer Bank (https://pga.mgh.harvard.edu/primerbank/) or were designed (Table 2). PCR reactions were run on BioRad CFX96 Real Time PCR instrument (BioRad Laboratories, CA, USA). The thermal cycling conditions were an initial polymerase activation at 92 °C for 12 minutes, followed by 40 cycles of denaturation at 95 °C for 15 seconds, annealing at 60 °C for 20 seconds and extension at 72 °C for 20 seconds. The melt curve protocol followed with 15 seconds at 95 °C and then 5 seconds each at 0.5 °C increment from 65 °C to 95 °C. The gene expression analysis was evaluated in three experiments. All reactions were run in triplicate. Gene expression data were normalized to ACTB (β-actin) housekeeping gene. Mean cycle threshold (Ct) values were estimated with BioRad CFX Manager 3.1 software. Relative expression levels were calculated using the 2−ΔCt formula.

**Table 2:** Primer sequences used in RT-qPCR.

Gene	Primer	Sequence	Amplicon size (bp)	Reference
*ACTB*	Forward	CTTCCTGGGCATGGAGTCC	112	Designed
	Reverse	ATCTTGATCTTCATTGTGCTG		
*ALPL*	Forward	GCTCCAGGGATAAAGCAGGT	122	UCSC Genome Browser
	Reverse	CGCCAGTACTTGGGGTCTTT		
*FTL*	Forward	CAGCCTGGTCAATTTGTACCT	114	Primer Bank
	Reverse	GCCAATTCGCGGAAGAAGTG		
*PP2A*	Forward	TTGGTGTCTAGAGCTCACCAGC	125	UCSC Genome Browser
	Reverse	TCCATGATTGCAGCTTGGTT		
*PTPN11*	Forward	CTGGTGTGGAGGCAGAAAAC	125	UCSC Genome Browser
	Reverse	GTGGGTGACAGCTCCATTTC		
*TFRC*	Forward	ACCATTGTCATATACCCGGTTCA	219	Primer Bank
	Reverse	CAATAGCCCAAGTAGCCAATCAT		

### Reactive oxygen species (ROS) test

ROS production in SPIONs treated cells was investigated using fluorogenic probes DCFDA/H2DCFDA - Cellular ROS Assay Kit (Abcam, UK), according to the manufacturer’s protocol. TCam-2 and SW1353 were seeded at density of 2 × 10^4^ cells and 3 × 10^4^ (respectively) per well on 96-well microplate (Falcon white/clear bottom plate, Corning, NY, USA), 8 wells per group. After 24 h incubation, the SPIONs were applied to cells in the complete growth medium to final concentration 0.1 mg/mL, 0.05 mg/mL, 0.025 mg/mL and 0.0125 mg/mL. SW1353 and TCam-2 cells were treated with SPIONs and incubated for 24 h. Subsequently, cells were washed with 1X buffer and stained with 100 µl of 25 µM DCFDA in 1X Buffer for 45 min. at 37 °C. After the incubation, cells were washed with 1X Buffer. The fluorescence was measured using Tecan Infinite M200 Pro microplate reader at excitation/emission = 485/535 nm, multiple reads per well (3 × 3 matrix), in a fluorescence top reading mode.

### Statistical analysis

Statistical analysis was performed using GraphPad Prism 8 (GraphPad Software Inc., CA, USA). Statistical significance of the differences between means of gene expressions was determined with the unpaired t-test. P values < 0.05 were considered statistically significant. In other experiments, one-way ANOVA statistical analysis was used to calculate the significance of the data, α = 0.05.

## Supplementary Information

Supplementary data v.2
