# Supplementary material for: Bioevaluation of superparamagnetic iron oxide nanoparticles (SPIONs) functionalized with dihexadecyl phosphate (DHP)
Source: Sci Rep. 2020 Feb 17;10:2725. doi: 10.1038/s41598-020-59478-2 (PMC7026144; doi:10.1038/s41598-020-59478-2)
Supplement: Supplementary file 1 — Supplementary data v.2 [file 41598_2020_59478_MOESM1_ESM.docx]

**Title: Bioevaluation of superparamagnetic iron oxide nanoparticles (SPIONs) functionalized with dihexadecyl phosphate (DHP).**

**(Supplementary information)**

**Authors:** Adam Aron Mieloch^1^, Magdalena Żurawek^2^, Michael Giersig^1,3^, Natalia Rozwadowska^2^ and Jakub Dalibor Rybka^1,^*

**^1^** Center for Advanced Technology, Adam Mickiewicz University, Uniwersytetu Poznańskiego 10 Street, 61-614 Poznan, Poland

**^2^** Institute of Human Genetics Polish Academy of Sciences, ul. Strzeszynska 32 60-470 Poznan, Poland

**^3^** Department of Physics, Institute of Experimental Physics, Freie Universität, Arnimallee 14, 14195 Berlin, Germany


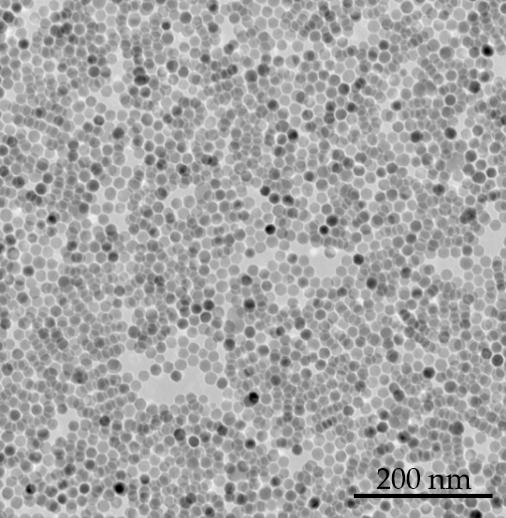


**A**

**B**

**Figure S1. A** - TEM image of superparamagnetic iron oxide nanoparticles (SPIONs) after the synthesis. **B** – Size distribution of as-synthesized SPIONs. Analyzed with ImageJ and GraphPad 8 Software.

**Figure S2.** Thermogravimetric analysis of SPION-DHP nanoparticles performed in triplicate. The point of the lowest mass was used for calculation of SPION-DHP concentration.
